# The Role of Processed *Aloe vera* Gel in Intestinal Tight Junction: An In Vivo and In Vitro Study

**DOI:** 10.3390/ijms22126515

**Published:** 2021-06-17

**Authors:** Thu Han Le Phan, Se Yong Park, Hyun Jin Jung, Min Woo Kim, Eunae Cho, Kyu-Suk Shim, Eunju Shin, Jin-Ha Yoon, Han-Joo Maeng, Ju-Hee Kang, Seung Hyun Oh

**Affiliations:** 1 College of Pharmacy, Gachon University, Incheon 21936, Korea; lephan.thuhan@gmail.com (T.H.L.P.); walkingjin86@gmail.com (H.J.J.); jinha89@daum.net (J.-H.Y.); hjmaeng@gachon.ac.kr (H.-J.M.); 2 College of Veterinary Medicine, Seoul National University, Seoul 08826, Korea; tpdyd2468@gmail.com (S.Y.P.); eastsea1203@gmail.com (M.W.K.); 3 Univera Co., Ltd., Seoul 04782, Korea; Eunaec@univera.com (E.C.); kssim93@univera.com (K.-S.S.); ejayshin@univera.com (E.S.)

**Keywords:** leaky gut, *Aloe vera* gel, tight junction, ZO-1, MAPK/ERK, 4E-BP1

## Abstract

Leaky gut is a condition of increased paracellular permeability of the intestine due to compromised tight junction barriers. In recent years, this affliction has drawn the attention of scientists from different fields, as a myriad of studies prosecuted it to be the silent culprit of various immune diseases. Due to various controversies surrounding its culpability in the clinic, approaches to leaky gut are restricted in maintaining a healthy lifestyle, avoiding irritating factors, and practicing alternative medicine, including the consumption of supplements. In the current study, we investigate the tight junction-modulating effects of processed *Aloe vera* gel (PAG), comprising 5–400-kD polysaccharides as the main components. Our results show that oral treatment of 143 mg/kg PAG daily for 10 days improves the age-related leaky gut condition in old mice, by reducing their individual urinal lactulose/mannitol (L/M) ratio. In concordance with in vivo experiments, PAG treatment at dose 400 μg/mL accelerated the polarization process of Caco-2 monolayers. The underlying mechanism was attributed to enhancement in the expression of intestinal tight junction-associated scaffold protein zonula occludens (ZO)-1 at the translation level. This was induced by activation of the MAPK/ERK signaling pathway, which inhibits the translation repressor 4E-BP1. In conclusion, we propose that consuming PAG as a complementary food has the potential to benefit high-risk patients.

## 1. Introduction

Intestinal tight junctions are complex structures containing various intercellular membrane proteins (such as claudins and occludin) attached to the scaffolding zonula occludens proteins (ZOs), and through them, to the cytoskeleton actin and myosin of intestinal epithelial cells. Together, they form a barrier that dictates the paracellular absorption of electrolytes and macro-molecules, thereby contributing to safeguarding our body against a bacteria-laden intestinal lumen [1]. First mentioned in the 1990s [2,3,4] but not later, leaky gut has recently garnered attention in scientific reports as a risk factor for various autoimmune diseases, subsequent to the discovery of bacterial compartments translocating to different organs and locations of the human body [5,6,7]. For leaky gut is assumed to originate due to compromised intestinal tight junction integrity [8,9], concerns have been raised to identify factors that undermine this integrity, and exercises have been undertaken to preserve the efficacy, including consuming beneficial types of food.

*Aloe vera* (L.) Burm.f. gel, a transparent viscous liquid derived from the leaf pulp of plants belonging to the family Asphodelaceae, is ubiquitously consumed by people with varying healthcare backgrounds for its anti-inflammatory, anti-oxidative, and healing properties [10,11]. Among the numerous phytochemical components of *Aloe vera* gel, polysaccharides are the mainstay in various therapeutic effects [12]. Several recent studies have reported on the effects of polysaccharides from different origins on tight junctions [13,14,15,16,17,18,19]. We therefore hypothesized that the polysaccharides of *Aloe vera* gel are likely to possess bioactivity to help alleviate the leaky gut condition. To our knowledge, the effects of *Aloe vera* gel on leaky gut have not been reported elsewhere. This work investigated whether our processed *Aloe vera* gel (PAG) could enhance the intestinal barrier integrity via the reinforcement of the tight junction. Our results provide empirical evidence to consider PAG consumption for individuals with distinct medical conditions that may be related to a leaky gut condition.

## 2. Results

### 2.1. PAG Improves Age-Related Leaky Gut Condition

**Figure 1:**
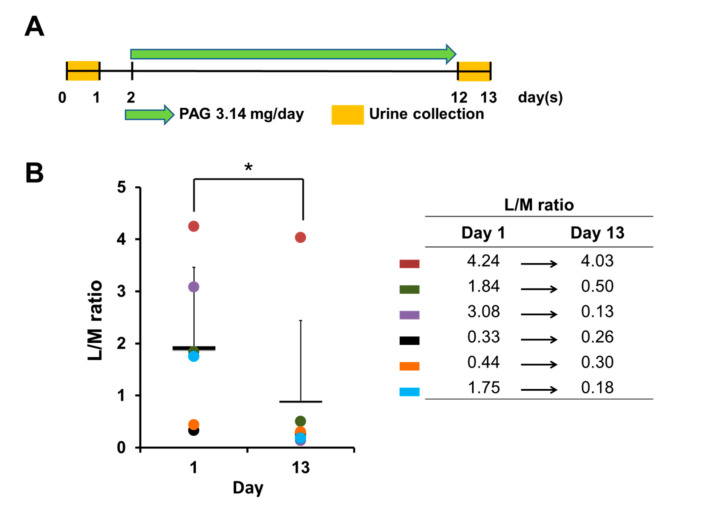
PAG improves leaky gut condition in vivo. (**A**) Experiment schedule: two-year-old C57BL/6 mice were treated with 143 mg/kg PAG daily for 10 days; 24-h urine samples were collected to determine lactulose and mannitol concentrations before and after PAG administration; (**B**) L/M ratio of mice before and after PAG treatment, presented as mean ± standard deviation (Student *t*-test was applied for statistical analysis, * *p* < 0.05); different colors represent different mice within the group.

To investigate whether PAG modulates the intestinal barrier, we employed a murine model of an age-related compromised intestinal barrier in which the intestinal permeability of old mice was assessed before and after 10 days of PAG administration, by evaluating the lactulose/mannitol (L/M) ratio in the 24-h urine (Figure 1A). In the L/M test, absorptive ability of the large molecule (lactulose) is minimal in the normal intestine and increases in cases of intestinal barrier breach, whereas the small molecule (mannitol) is always absorbed proportionally to the intestinal up-take capacity [20]. Therefore, any decrease in the L/M ratio serves as a marker of intestinal integrity improvement. Our results reveal that PAG consumption successfully decreases the urinal L/M ratio of all old mice after 10-day treatment (Figure 1B), indicating that PAG treatment significantly improves the intestinal barrier in old mice.

### 2.2. PAG Increases Tight Junction Proteins of Intestinal Epithelial Cells

**Figure 2:**
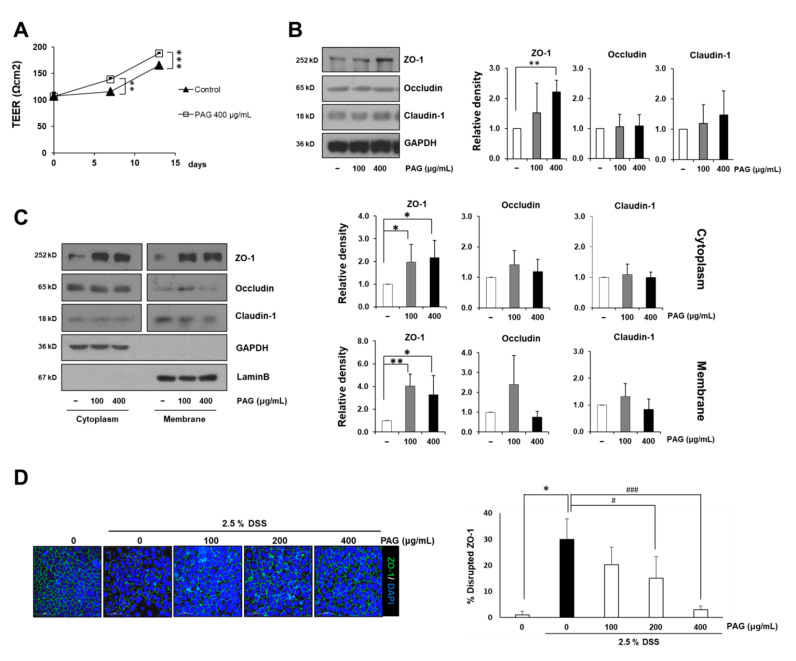
PAG reinforces the intestinal barrier in vitro by increasing expression levels of tight junction proteins. (**A**) Caco-2 monolayers were cultured with or without PAG 400 μg/mL for 13 days, and trans-epithelial electric resistance values of the monolayers were recorded at days 0, 7, and 13 in triplicate; (**B**,**C**) Expression levels of tight junction-related proteins in total cell lysate (**B**), cytoplasmic (soluble) fraction and cytomembrane (insoluble) fraction (**C**) of Caco-2 monolayers after 48-h incubation with varying concentrations of PAG; (**D**) 14-day polarized Caco-2 monolayers were incubated with or without 2.5% DSS for 48 h after pre-treatment with PAG 400 μg/mL for 24 h; immunostaining was subsequently performed to visualize expression levels of the ZO-1 protein. Values are presented as mean ± standard deviation (Student *t*-test was applied for statistical analysis; *, # *p* < 0.05; ** *p* < 0.01; ***, ### *p* < 0.001).

The human intestinal epithelial cell line Caco-2 is broadly used to investigate the integrity of the intestinal barrier, due to its ability to form a monolayer containing intercellular tight junctions when reaching 100% confluency [21]. In the current study, fully confluent Caco-2 monolayers were treated with or without 400 μg/mL of PAG for 13 days, and their trans-epithelial electrical resistance (TEER) values were recorded before, in the middle, and at the end of the experiment. During the experimental period, the TEER values of Caco-2 monolayers increase gradually, indicating tight junction formation of the cells. PAG treatment accelerate this process as the PAG-treated monolayer always obtained higher TEER values compared with its control counterpart (Figure 2A). Immunoblotting with Caco-2 whole cell lysates reveals that two typical tight junction-related proteins, occludin and claudin-1, fluctuate subsequent to PAG exposure at doses 100 and 400 μg/mL, whereas ZO-1 shows remarkable increases (Figure 2B). Although ZO-1’s changes under the low dose are insignificant, PAG seems to induce dose-dependent increases in ZO-1 expression levels.

Next, we fractionated the cytoplasmic (soluble) and membranous (insoluble) fractions of Caco-2 cells to examine whether increased ZO-1 reflects the fortification of tight junctions which connect the adjacent cell membranes. In concordance to the whole cell lysate results, under PAG treatment, occludin and claudin-1 proteins do not register any significant changes in both fractions, confirming the disassociation between PAG’s effects and these two tight junction-related proteins. Interestingly, we found that exposure to PAG significantly increases the expression levels of ZO-1 in both fractions of non-polarized Caco-2 monolayers (Figure 2C). The fully differentiated Caco-2 cells were employed to mimic leaky gut condition in vitro, in which tight junction was damaged by DSS 2.5%. Immunostaining with ZO-1 specific antibody confirmed the protective effects of PAG against DSS-induced intestinal tight junction disruption in vitro. In contrast to the discontinuous and damaged ZO-1 barrier of the DSS-treated Caco-2 monolayer, the PAG-treated samples remained comparatively intact and uninterrupted (Figure 2D). Taken together, our results indicate that the ability of PAG to enhance intestinal tight junction-related scaffold protein ZO-1 underlies its intestinal barrier preserving activities.

### 2.3. PAG Conserves Intestinal Tight Junction at Translation Level by Deactivating 4E-BP1 via 115 MAPK/ERK Signaling Pathway 116

**Figure 3:**
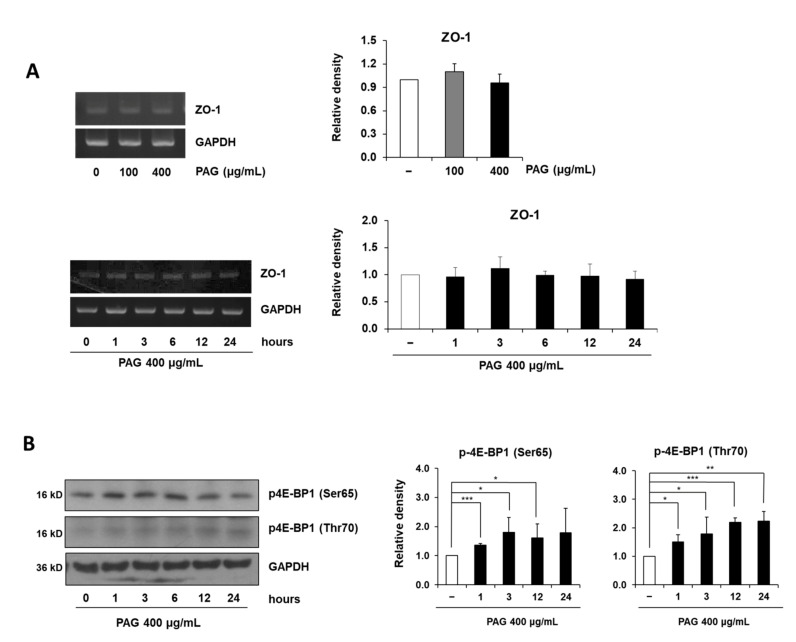
PAG increases expression level of ZO-1 at translation level. (**A**) Dose-dependent and time-dependent alterations of ZO-1 at the mRNA level in Caco-2 monolayers treated with 400 μg/mL PAG; (**B**) PAG 400 μg/mL exposure time-dependently induces phosphorylation of the translation repressor 4E-BP1 and ERK1/2 in Caco-2 monolayers. Values are presented as mean ± standard deviation (Student *t*-test was applied for statistical analysis, * *p* < 0.05, ** *p* < 0.01, *** *p* < 0.001).

Despite the significant increase in ZO-1 protein expression levels subsequent to PAG treatment, no alteration was observed at the RNA level, in either the dose-dependent or time-dependent manner (Figure 3A). This suggests that PAG regulates the ZO-1 expression via a non-transcriptional process. Recently, we found that PAG treatment time-dependently increases phosphorylation at the Thr70 and Ser65 positions of 4E-BP1 (Figure 3B), an inhibitor of eukaryotic initiation factors which represses translation, with different patterns. Although increasing 4E-BP1 phosphorylation at both positions were initiated one hour after PAG treatment, the 4E-BP1 phosphorylation at Ser65 was maintained at a similar level for over 12 h before decreasing to the basal level 24 h after exposure, whereas the 4E-BP1 phosphorylation at Thr70 registered a gradual increase during 24 h of treatment.

**Figure 4:**
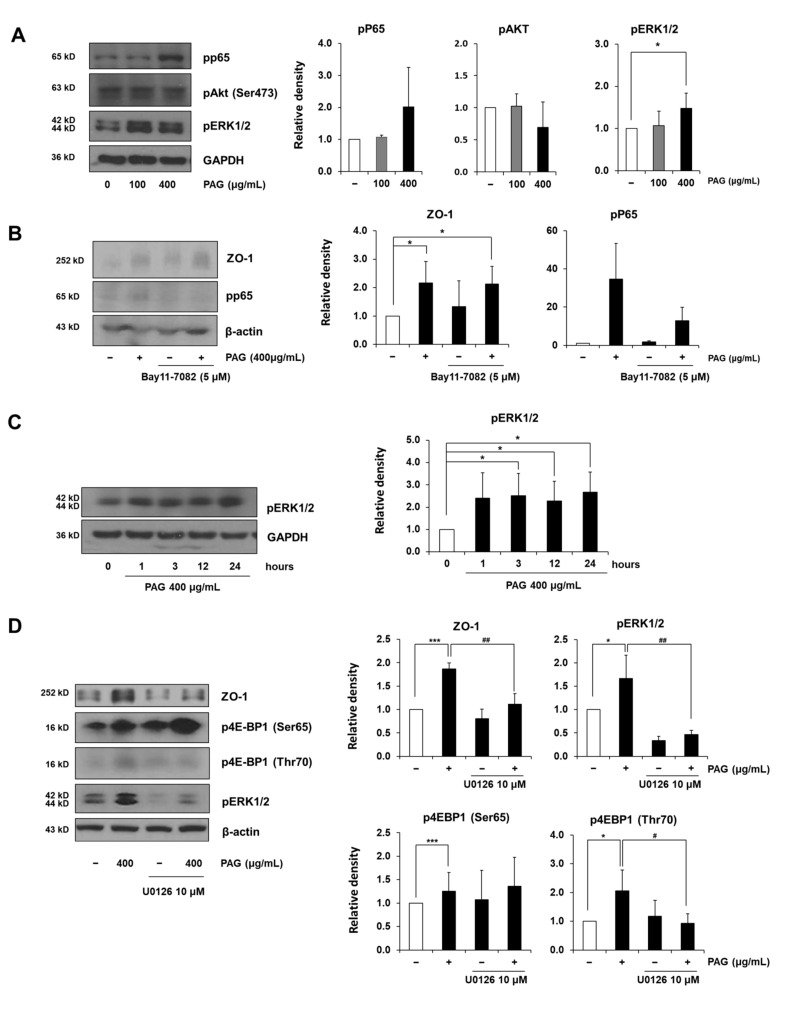
PAG increases expression level of ZO-1 via activating MAPK/ERK signaling pathway. (**A**) Caco-2 monolayers were treated with PAG to investigate different tight junction-related signaling pathways; (**B**) NF-κB inhibitor BAY11-7082 was employed to investigate the association between NF-κB/P65 and ZO-1 expression levels in Caco-2 cells under 48-h PAG treatment; (**C**) Caco-2 treated with PAG showed increased phosphorylated MAPK/ERK, in a pattern similar to phosphorylation of 4E-BP1; (**D**) Caco-2 monolayers were pre-treated with or without MAPK inhibitor U0126 10 μM along with exposure to PAG for 48-h, to determine the relation between MAPK/ERK, 4E-BP1, and ZO-1 expression levels. Values are presented as mean ± standard deviation (Student *t*-test was applied for statistical analysis; *, # *p* < 0.05; ## *p* < 0.01; *** *p* < 0.001).

**Figure 5:**
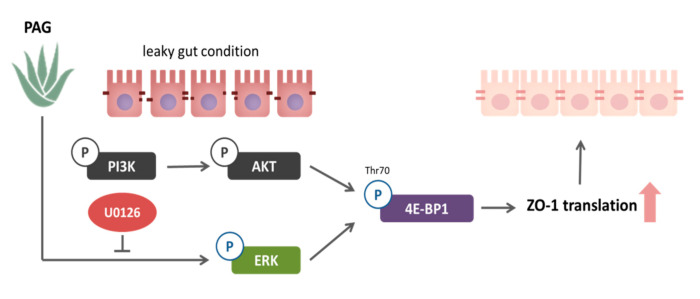
Schematic representation of the mechanism via which PAG regulates the intestinal barrier. In leaky gut sufferers administered PAG, the MAPK/ERK signaling pathway is activated in the intestinal epithelial cells, leading to Thr70-specific phosphorylation of the translation repressor 4E-BP1, resulting in a translational increase in the tight junction related protein ZO-1, to finally reinforcing the intestinal tight junction.

To determine which signaling pathway orchestrating ZO-1-induction is affected by PAG, we investigated the phosphorylated status of different proteins related to the tight junction and 4E-BP1 regulation, such as p65, ERK1/2, and AKT. Although AKT is the well-known 4E-BP1 regulator [22,23,24], no changes were observed in its phosphorylation after 72-h treatment with PAG 400 μg/mL (Figure 4A). Since PAG activates p65 and ERK1/2, we next treated Caco-2 monolayers with their corresponding activation inhibitors, viz., BAY11-7082 and U0126, respectively. Figure 4B shows that in concordance with previous results, although PAG induces increased phosphorylation of p65, this change is insignificant. Although 5 μM BAY11-7082 successfully blocks the PAG-induced activation of p65, there was no change in the expression level of the ZO-1 protein in cells treated with a combination of PAG and BAY11-7082, as compared to PAG-only treatment. Therefore, we concluded that the activation of the NF-κB pathway and the increase in the ZO-1 expression level are not associated. Conversely, we observed that along with the time-dependent increase in 4E-BP1 phosphorylation, subsequent to PAG exposure, phosphorylated ERK1/2 also revealed a similar pattern (Figure 4C), thereby suggesting that these two phenomena are associated for regulating the ZO-1 expression. To confirm this hypothesis, further experiments were conducted using 10 μM of ERK inhibitor (U0126) in combination with PAG treatment. Figure 4D shows that 10 μM U0126 does not exert any detrimental activities on the tight junction-related protein ZO-1 and the phosphorylation of the translation repressor 4E-BP1 under normal conditions, but successfully inhibits the increases in phosphorylated ERK and the tight junction related protein ZO-1 induced by PAG in Caco-2 monolayers, thereby confirming the correlation between the stimulation of the MAPK/ERK signaling pathway and the increased ZO-1 expression. This process was mediated at the translation level by the inhibition of 4E-BP1 via Thr70-specific phosphorylation, as U0126 treatment only abrogated the PAG-induced phosphorylation at this position without any impact on the Ser65-phosphorylated form (Figure 4D). In conclusion, our results indicate that exposure to PAG reinforces the intestinal tight junction related protein ZO-1 at the translation level by regulating the MAPK/ERK signaling pathway, thereby inhibiting the translation repressor 4E-BP1 via specific phosphorylation at Thr70. A schematic diagram of this mechanism is delineated in Figure 5.

## 3. Discussion

Accounting for about 10% by dry weight with an average molecular weight of 2 million kDa, polysaccharides are the main components contributing to the high viscosity of fresh *Aloe vera* gel [25]. Our *Aloe vera* gel was processed through different step to yield polysaccharides of 5 to 400 kDa and eliminate anthraquinones and colored substances which can trigger diarrhea [25]. The smaller polysaccharides decrease the stickiness of *Aloe vera* gel and facilitate its absorption; moreover, these changes in the polysaccharide profile could also bestow different therapeutic activities on PAG [25] besides the familiar anti-inflammatory, anti-oxidative, and healing properties. Previously, Kim et al. reported the effects of PAG in mucin secretion on intestinal goblet cells [26], proposing that efficacy of PAG on other intestinal epithelial cell types, which would subsequently strengthen the intestinal tight junction. A recent study of Na et al. revealed that oral PAG enhances tight junctions in the skin [27]; this further supports our hypothesis that PAG has the potential to alleviate the leaky gut condition.

Although not directly integrating the apical surfaces of adjacent intestinal epithelial cells, the peripheral membrane protein ZO-1 plays an important role in assembly and maintenance of the tight junction complex by functioning as a bridge between other transmembrane tight junction proteins (such as claudins and occludin) and various signaling pathways [28]. Various bioactivities of *Aloe vera* gel and its components are involved in the mitogen-activated protein kinase (MAPK) pathways [26,29,30] and MAPK is well-known to regulate the expression of different tight junction proteins [31]. Our results indicate activation of the MAPK/ERK regulated ZO-1-induction effect of PAG.

Despite a significant increase in ZO-1 protein levels after exposure of Caco-2 cells to PAG, the amount of mRNA is kept constant, thereby demanding investigation of either the translation level or degradation pathway. In one experiment, cycloheximide, a popular protein synthesis inhibitor in eukaryotes, was employed to determine whether PAG prevents the ZO-1 degradation. However, relatively high concentrations (20 and 50 μM) could only block the translation of other tight junction related proteins, such as occludin or claudin-1, but not ZO-1 (data not shown), suggesting that ZO-1 is a fairly stable protein. In another experiment, inactive forms of the translation repressor 4E-BP1 were examined, as it was reported to be under the control of MAPK/ERK [32,33,34,35]. Deactivation of 4E-BP1 occurs by phosphorylation at two critical positions: Thr70 and Ser65 [36,37,38]. Our study revealed that, in contrast with the subtle and ephemeral changes of the Ser65-phosphorylated form, Thr70-phosphorylated 4E-BP1 was significantly and continuously increased over a 24-h period of PAG treatment. Abrogation of this effect using the ERK inhibitor (U0126) finally built a bridge, linking the activation of the MAPK/ERK signaling pathway and increasing the ZO-1 expression subsequent to PAG exposure.

Leaky gut results from various stressful conditions and is associated with a myriad of diseases and disorders in different organs and functional systems [9]. Therefore, the ability of PAG to enhance the intestinal barrier integrity benefits a wide range of patients suffering from different diseases, people at risk, and even the normal population. However, numerous papers have reported *Aloe vera* polysaccharides as potential drug enhancers [39,40,41,42]. These effects were claimed to be attributed to the opening of tight junctions, which disputes our results. This contradiction can be explained by two reasons. First, differences in *Aloe vera* gel processing methods could alter the properties and biological activities of *Aloe vera* gel polysaccharides. In our study, the *Aloe vera* gel was processed to yield polysaccharides of 5–400 kDa, which are much smaller than the average 2000-kDa native *Aloe vera* gel used in other drug-enhancing studies. A study in 2005 by Im et al. reported the molecular size-activity relationship involved in the immunomodulatory effects of *Aloe vera* polysaccharides [25], which could exemplify this speculation. Secondly, doses of *Aloe vera* gel used in the drug enhancing studies were over 12 to 500 times higher than our doses (0.5–5%, i.e., 5–50 mg/mL, as compared with 0.1–0.4 mg/mL, respectively). Therefore, tight junction openings could result from the destructive effects of such high doses applied. Although it raises concern about how much *Aloe vera* gel intake can inflict intestinal barrier breach and exacerbate, to date there are no studies showing that *Aloe vera* gel increases the intestinal permeability in vivo and in humans. Despite the effects of *Aloe vera* gel to enhance human absorption of vitamin C and E, the mechanisms were presumed to be due to the ability of *Aloe vera* gel to prevent gastric degradation, and bind to the vitamins and slow-down their absorption [43]. Moreover, in 2004, a randomized, double-blind, placebo-controlled study of Langmead et al. offered evidence that oral intake of *Aloe vera* gel 100 mL twice daily for four weeks safely induces clinical remission, clinical improvement, and clinical response, as well as improved histological scores of ulcerative colitis—a disease directly associated with compromised intestinal barrier [44,45,46,47], in out-patients [48].

## 4. Materials and Methods

### 4.1. Animals, Cells, and Materials

Male and female two-year-old mice were supplied by the Korea Research Institute of Bioscience and Biotechnology (KRIBB—Ochang, Cheongwon, Chungbuk, Korea). All animal procedures were conducted according to the protocol approved by the Institutional Animal Care and Usage Committee [GIACUC-R2020019] at Gachon University in Incheon, Korea. A Caco-2 human colon carcinoma cell line was obtained from the Korea Cell Line Bank (Seoul, Korea). Cells were cultured in DMEM medium supplemented with 10% fetal bovine serum and penicillin/streptomycin (Welgene, Inc., Daegu, Korea). The ZO-1 (61-7300), occludin (33-1500), and claudin-1 (37-4900) antibodies were purchased from Invitrogen (Carlsbad, CA, USA). The antibody against lamin B (sc-6217) was purchased from Santa Cruz Biotechnology (Santa Cruz, CA, USA). The phospho-ERK1/2 (#4370), phospho-AKT (#9271), phospho-4e-BP1(Ser65, #9451), and phospho-4e-BP1 (Thr70, 9455) antibodies were purchased from Cell Signaling Technology (Danvers, MA, USA). The GAPDH (CB-1001) and β-actin (SC-1615) antibody was purchased from Sigma-Aldrich (St. Louis, MO, USA). PAG was provided by Univera Co., Ltd. (Seoul, Korea). The *Aloe vera* gel underwent grinding and depulping steps, followed by charcoal filtering to remove anthraquinones and other colored substances, before being concentrated and dried. Determination of the total polysaccharide content and molecular weight distribution of polysaccharides in PAG were performed, as previously described [25].

### 4.2. Assessment of the Intestinal Barrier—L/M Test and Teer Measurement

The L/M test was conducted as described previously [49]. During the experiment, mice had ad libitum access to water. All mice were starved for six hours before oral administration of 250 µL solution containing 20 mg/mL lactulose and 50 mg/mL mannitol. After gavage, mice were fasted for one hour before re-feeding, and their urine was subsequently collected using metabolic cages over a 24 h period. Collected urine samples were analyzed for sugar concentration using the liquid chromatography–mass spectrometry (LC/MS) system consisting of an Agilent 6495 Triple Quadrupole LC/MS coupled with an Agilent Technologies 1260 HPLC system (Agilent Technologies, Santa Clara, CA, USA). To separate the endogenous substances from these sugars in urine, a Luna amino column (150 mm × 2 mm ID, 3 μm particle size; Phenomenex, Torrance, CA, USA) was used at a flow rate of 0.2 mL/min, as described previously [50]. The mobile phase was a mixture of water (solvent A) and ACN (solvent B), using a gradient program (80 *v*/*v*% solvent B for 1 min, 80 to 20 *v*/*v*% solvent B over 4 min, 20 *v*/*v*% solvent B for 3 min, 20 to 80 *v*/*v*% solvent B over 0.1 min, and 80 *v*/*v*% solvent B for 6.9 min). Concentration levels were calculated by applying the Mass Hunter software (version A.02.00; Agilent Technologies, Santa Clara, CA, USA). In the negative electrospray ionization (ESI) mode, the MRM transitions of lactulose, mannitol, and an internal standard (IS), salicin, were as follows: 341.1→160.9, 181.0→89.0, and 5.1→123.0, respectively. For sample preparation, the collected mouse urine was diluted 10-fold with purified water, and 100 μL aliquots were added to 200 μL acetonitrile containing IS (25 μg/mL). After centrifugation for 5 min at 14,000 rpm, the supernatant was separated and subjected to the LC-MS/MS system. The calibration ranges obtained were from 1 to 20 μg/mL with a good correlation (R > 0.999). The urinary recovery of lactulose and mannitol was calculated as percentage of dose (% dose).

To investigate intestinal barrier integrity in vitro, Caco-2 cells were seeded onto cell culture inserts (Transwell, Corning-Costar, Corning, NY, USA) and rested until fully confluent. Once a day the Caco-2 monolayers were treated with or without 400 μg/mL PAG for 13 days and their TEER values were recorded at days 0, 7, and 13 of the experiment, using an EVOM2 chopstick electrode and EVOM2 Epithelial Volt/Ohm meter (World Precision Instruments, Sarasota, FL, USA). The TEER measurement was performed in triplicate and the averages were employed to assess the activity of PAG to reinforce the tight junction barrier.

### 4.3. Western Blot Analysis

Caco-2 cells were seeded 1.5 × 10^6^ cells/well in a 6-well plate and rested for 48 h to attain 100% confluency. Caco-2 monolayers were then treated with or without 100 μg/mL or 400 μg/mL PAG for indicated times. For cell fractionation, the cytoplasm proteins (detergent soluble fraction) were dissolved in Triton X-100 lysis buffer, and the membrane proteins (detergent insoluble fraction) were dissolved in sodium dodecyl sulfate (SDS) lysis buffer. For total cell lysate, cell proteins were isolated using RIPA buffer. Protein concentrations were determined using the bicinchoninic acid protein assay (Pierce Biotechnology, Rockford, IL, USA). Briefly, in a 96-well plate, each cell lysate sample was diluted with water in a 2:23 ratio, followed by reacting with 200 µL BCA working reagent, made from mixing BCA A and B solutions in a 1:50 ratio. The plate was then incubated at 37 °C for 25 min, followed by 10-min rest at room temperature, before the absorbance value of each well was measured at a 560 nm wavelength, using a spectrophotometer (Thermo Scientific Multiskan GO, Thermo Fisher Scientific, Waltham, Massachusetts, USA). The concentrations of protein samples were calculated by referring to a linear standard curve constructed from varying concentrations (0–1000 μg/mL) of bovine serum albumin (BSA). Finally, protein samples were mixed with autoclaved distilled water and sample buffer to yield the same concentration and denatured in a heat block at 95 °C for 5 min. SDS-polyacrylamide gel electrophoresis was performed with an equal amount of each blotted protein sample (40 μg/well), and the membrane-binding antibodies were detected using enhanced chemiluminescence western blotting detection reagents obtained from Absignal (Abclone, Seoul, Korea).

### 4.4. Polymerase Chain Reaction (PCR)

Gene expression changes at the RNA level were determined using a reverse transcriptase (RT)-polymerase chain reaction (PCR). Briefly, total RNA was isolated using the TRIzol reagent (Invitrogen, Carlsbad, CA, USA), followed by complementary DNA (cDNA) synthesis from 2 μg RNA using the PrimeScript RT reagent Kit (TaKaRa, Shiga, Japan). The cDNA from Caco-2 cells was then amplified by PCR with human-specific primers: ZO-1, 5′-AAC GCT ATG AAC CCA TCC AG-3′ (forward) and 5′-CGG TTT GGT GGT CTG AAA GT-3′ (reverse); and GAPDH, 5′-G GTG AAG GTC GGT GTG AAC GGA TTT-3′ (forward) and 5′-AAT GCC AAA GTT GTC ATG GAT GAC C-3′ (reverse). After PCR, the resultant products were analyzed by separation on a 1.5% agarose gel in tris-acetate/ethylenediamine-tetraacetic acid (EDTA) buffer.

### 4.5. Immunofluorescence Assay

To evaluate the effect of PAG on DSS-treated Caco-2, an immunofluorescence assay was performed as previously described [51]. Briefly, cells were seeded onto coverslips placed in 12-well plates (Costar, Corning, NY, USA), and allowed to polarize for 14 days before pre-treatment with PAG for 24 h, and subsequent incubation with or without 2.5% DSS for 48 h. Next, the cells were fixed and permeabilized in a methanol–acetone (1:1) mixture at −20 °C for 20 min, followed by overnight incubation with ZO-1 primary antibodies at 4 °C, and subsequent incubation with an FITC-labeled secondary antibody for 2 h at room temperature. Coverslips were then mounted with mounting medium containing 4,6-diamidino-2-phenylindole (DAPI) for nuclear counterstaining. FITC and DAPI images were taken from the same area. The intact of ZO-1 barriers were assessed under the microscope by calculating average disruption levels at 4 different areas of each sample.

### 4.6. Statistical Analysis

Statistical analysis was conducted using Microsoft Excel, assuming that population distributions of each group have equal variances. Independent *t*-test or/and paired *t*-test were performed to compare means of histological scores and L/M ratios, respectively. All *t* Stat values are within their corresponding *t* Critical ranges and one-tail *p* values were registered.

## 5. Conclusions

In conclusion, our data determines that PAG enhances the intestinal tight junction related protein ZO-1 through activation of the MAPK/ERK signaling pathway, to inhibit translation repressor 4E-BP1 by specific phosphorylation at its Thr70. Our study also proposes that PAG consumption could benefit people who are suffering from or at risk of leaky-gut condition. Further studies are required to assess the protective effects of PAG on different models of compromised intestinal tight junction integrity-related diseases.

## Data Availability

The data that support the findings of this study are available from the corresponding author upon reasonable request.
